# A Novel Neighborhood-Based Computational Model for Potential MiRNA-Disease Association Prediction

**DOI:** 10.1155/2019/5145646

**Published:** 2019-01-17

**Authors:** Yang Liu, Xueyong Li, Xiang Feng, Lei Wang

**Affiliations:** ^1^Key Laboratory of Hunan Province for Internet of Things and Information Security, Xiangtan University, Xiangtan, China; ^2^College of Computer Engineering and Applied Mathematics, Changsha University, Changsha 410001, China

## Abstract

In recent years, more and more studies have shown that miRNAs can affect a variety of biological processes. It is important for disease prevention, treatment, diagnosis, and prognosis to study the relationships between human diseases and miRNAs. However, traditional experimental methods are time-consuming and labour-intensive. Hence, in this paper, a novel neighborhood-based computational model called NBMDA is proposed for predicting potential miRNA-disease associations. Due to the fact that known miRNA-disease associations are very rare and many diseases (or miRNAs) are associated with only one or a few miRNAs (or diseases), in NBMDA, the *K*-nearest neighbor (KNN) method is utilized as a recommendation algorithm based on known miRNA-disease associations, miRNA functional similarity, disease semantic similarity, and Gaussian interaction profile kernel similarity for miRNAs and diseases to improve its prediction accuracy. And simulation results demonstrate that NBMDA can effectively infer miRNA-disease associations with higher accuracy compared with previous state-of-the-art methods. Moreover, independent case studies of esophageal neoplasms, breast neoplasms and colon neoplasms are further implemented, and as a result, there are 47, 48, and 48 out of the top 50 predicted miRNAs having been successfully confirmed by the previously published literatures, which also indicates that NBMDA can be utilized as a powerful tool to study the relationships between miRNAs and diseases.

## 1. Introduction

MiRNAs are one kind of small RNAs with the length of about 20–24 nucleotides that can regulate the expression of posttranscriptional genes, and each miRNA may have multiple target genes that can be regulated by multiple miRNAs as well [1–4]. Recently, more and more studies have shown that miRNAs play important roles in many physiological processes of the human body such as cell growth [5], proliferation [6], differentiation [7], immune response [8] embryonic development [5], and so on. In addition, emerging evidences have implied as well that miRNAs can affect the occurrence and development of various tumors by regulating the signaling pathways in which their target genes are involved and play a role similar to oncogenes or tumor suppressor genes [9]. For example, miR-203 can inhibit the formation of esophageal tumors [10], miR-328 is a key oncogene in hepatocellular carcinoma, and its expression level will be significantly upregulated and downregulated in hepatocellular carcinoma tissues [11]. MiR-143 and miR-145 are expressed at low levels in esophageal cancer and gastric cancer, which mean that the downregulation of these two kinds of miRNAs can be considered as a potential biomarker for related tumors [12]. Hence, the exploration of potential relationships between miRNAs and diseases will have important significance for disease prevention, treatment, diagnosis, and prognosis [13–15].

Up to now, many human miRNA-disease association databases such as HMDD [16] and miR2Disease [17] have been established, in which the stored associations are mainly collected from previous biological experiments. And with the rapid growth of known biological information associated with miRNAs and diseases, known miRNA-disease associations are becoming far from meeting the needs of modern medical researches, since traditional methods of detecting miRNA-disease associations (e.g., PCR [18] and northern blotting [19]) are very time-consuming and labour-intensive. Therefore, in recent years, a large number of computational models have been proposed [20–22]. For instance, based on the assumption that similar miRNAs tend to be related or unrelated with similar diseases [23], Zeng et al. [24] proposed a model to infer potential associations between miRNAs and diseases based on the miRNA similarity, disease similarity, and known miRNA-disease associations. Yu et al. [25] proposed a model called KATZMDA to predict potential miRNA-disease associations by measuring the number and length of paths existing between a pair of miRNA-disease nodes in the miRNA-disease association network. In addition, considering that more and more biological databases have been established by far, it is obvious that the prediction performance would be improved more effectively, if more information collected from more databases are integrated to predict potential miRNA-disease associations. For example, Yu et al. [26] proposed a model called NBCLDA to infer potential associations between lncRNAs and diseases through integrating known miRNA-disease associations, miRNA-lncRNA associations, lncRNA-disease associations, gene-lncRNA associations, gene-disease associations, and gene-miRNA associations. Moreover, for the past few years, with machine learning gradually becoming a hot topic in many fields, some machine learning algorithms have been adopted to predict miRNA-disease associations as well. For instance, Zhang et al. [27] proposed a semisupervised model to infer potential miRNA-disease associations by implementing label propagation algorithms on the miRNA-disease association network. Chen et al. [28] proposed a computational model called SDMMDA based on super-diseases and miRNAs to predict potential miRNA-disease associations, in which as many as possible similar diseases or miRNAs would be clustered into super-diseases or super-miRNAs first, and then the Naive Bayesian scheme was utilized to infer potential associations between miRNAs and diseases. Luo et al. [29] proposed a semisupervised method called KRLSM to identify potential miRNA-disease associations, in which, due to the sparsity of known miRNA-disease associations, different omics data were integrated to improve the prediction accuracy of KRLSM first, and then, the semisupervised classifier of regularized least squares was adopted to calculate the potential probabilities of associations between miRNAs and diseases.

In this paper, different from above mentioned models, a novel neighborhood-based computational model called NBMDA was developed to infer potential miRNA-disease associations, in which, due to the fact that known miRNA-disease associations are quite sparse and there are a variety of diseases (or miRNAs) associating with only one or few miRNAs (or diseases), the *K*-nearest neighbor (KNN) method would be utilized as a recommendation algorithm to improve the prediction accuracy of NBMDA first, and then, according to two kinds of newly constructed miRNA-disease association networks and the original miRNA-disease association network, the possibilities of potential associations between miRNAs and diseases would be calculated based on the concept of common neighbors. Finally, in order to evaluate the prediction performance of NBMDA, global leave-one-out cross validation (global LOOCV) and 5-fold cross validation (5-fold CV) were implemented simultaneously, and simulation results demonstrated that NBMDA could achieve reliable AUCs of 0.8983/0.8153 and 0.8975 under the global LOOCV and 5-fold CV, respectively, which were higher than several state-of-the-art computational models. In addition, we further implemented the case studies of esophageal neoplasms, breast neoplasms and colon neoplasms on NBMDA, and simulation results illustrated that there were 47, 48, and 48 out of the top 50 predicted miRNAs having been successfully confirmed by the previously published literatures separately, which also demonstrated that NBMDA has good performance in predicting potential miRNA-disease associations. Hence, it is obvious that NBMDA can be further applied to predict both diseases without any known related miRNAs and miRNAs without any known related diseases.

## 2. Materials and Methods

### 2.1. Human miRNA-Disease Associations

In order to evaluate the performance of our proposed NBMDA, we use two datasets. The first dataset (denoted as dataset1) was downloaded from the HMDD v2.0 database, which consisted of 5430 experimentally validated human miRNA-disease associations including 495 different miRNAs and 383 different diseases [16]. The second dataset (denoted as dataset2) was downloaded from the miR2Disease database and the HMDD database, which consisted of high-quality experimentally verified microRNA-disease associations [14, 30]. As for the dataset2, after deleting 13 miRNAs that could not be found in the website http://www.cuilab.cn/files/images/cuilab/misim.zip, we finally obtained 250 miRNA-disease associations including 105 different miRNAs and 52 different diseases. And for convenience, we adopted an adjacency matrix *A* to represent the miRNA-disease associations, in which, for any given disease *d*(*i*) and miRNA *m*(*j*), if there is a known association between them, then the value of *A*(*i*, *j*) will be set to 1, otherwise *A*(*i*, *j*) will be set to 0. Therefore, the *i*th row of *A* denotes the interaction profiles of the disease *d*(*i*) with each of these collected miRNAs, and the *j*th column of *A* indicates the interaction profiles of the miRNA *m*(*j*) with each of these collected diseases. And moreover, the number of diseases and miRNAs collected in this paper will be represented by *N*_*d*_ and *N*_*m*_, respectively. Hence, based on the adjacency matrix *A*, we can obtain an original miRNA-disease association network MDA.

### 2.2. miRNA Functional Similarity

The miRNA functional similarity network can be established based on the assumption that functionally similar miRNAs are always associated with similar diseases [31]. In this section, in order to construct the miRNA functional similarity network, we downloaded the functional similarity scores between miRNAs collected in this study from the website http://www.cuilab.cn/files/images/cuilab/misim.zip and then, for convenience, we use *FS* to represent the miRNA functional similarity matrix, in which, the value of *FS*(*i*, *j*) represents the similarity score between the miRNA *m*(*i*) and the miRNA *m*(*j*).

### 2.3. Disease Semantic Similarity

The association between diseases can be represented by an directed acyclic graphs (DAGs), in which, a disease *D* can be described as DAG(*D*) = (*D*, *T*(*D*), *E*(*D*)), where *T*(*D*) represents a set of nodes including the *D* itself and its all ancestor nodes and *E*(*D*) is a set consisting of direct edges that connect parent nodes and child nodes in *T*(*D*). And moreover, the contribution of a disease *d* to the semantic value of *D* can be calculated according to the following formula:

(1) $$\begin{array}{c} \left\{\begin{array}{c} D_{\text{D}}\left(d\right)=1,\;\text{if}\;\;d=D,\\ D_{\text{D}}\left(d\right)=\max\left\{\left\{\Delta_{*}D_{\text{D}}\left(d^{\prime }\right)\left|d^{\prime }\right.\in \text{children}\;\;\mathrm{of}\;\;d\right\}\right\},\;\text{if}\;\;d\neq D. \end{array}\right. \end{array}$$

Additionally, the semantic value of disease *D* can be obtained as follows:

(2) $$\begin{array}{c} \text{DV}\left(D\right)=\underset{d\in T\left(D\right)}{\sum }D_{\text{D}}\left(d\right). \end{array}$$

Here, ∆ is a semantic contribution factor between 0 and 1, which will be set to 0.5 in this paper according to related works [32, 33]. And according to the above Formula (1), it is easy to see that the contribution of the disease *D* to the semantic value of itself is 1 and the contribution of an ancestor disease *d* to the semantic value of *D* gradually decreases with the increasing of the distance between them, which is regulated by ∆. And additionally, according to Formula (2), it is obvious that the semantic value of *D* is the sum of the contributions of ancestor diseases to the semantic values of *D*. In general, based on the assumption that if two diseases share more parts of the DAGs, there should be a higher semantic similarity between them, and the semantic similarity between the disease *d*(*i*) and *d*(*j*) can be obtained according to the following formula:

(3) $$\begin{array}{c} SS\left(d\left(i\right),d\left(j\right)\right)=\frac{\sum_{t\in T\left(d\left(i\right)\right)\cap T\left(d\left(j\right)\right)}\left(D_{d\left(i\right)}\left(t\right)+D_{d\left(j\right)}\left(t\right)\right)}{\text{DV}\left(d\left(i\right)\right)+\text{DV}\left(d\left(j\right)\right)}. \end{array}$$

Thereafter, according to Formula (3), we can obtain a *N*_*d*_ × *N*_*d*_ dimensional disease semantic similarity matrix *SS* based on these *N*_*d*_ diseases collected previously.

### 2.4. Gaussian Interaction Profile Kernel Similarity for miRNAs and Diseases

In this section, based on the hypothesis that similar miRNAs are always related or unrelated to similar diseases, we will adopt the topological information of known miRNA-disease association network to calculate the Gaussian interaction profile kernel similarity for miRNAs. Firstly, let the binary vector IP(*m*(*i*)) indicate the *i*th column of the adjacency matrix *A*, then, the Gaussian kernel similarity between the miRNA *m*(*i*) and the miRNA *m*(*j*) can be obtained according to the following formula:

(4) $$\begin{array}{c} KM\left(m\left(i\right),m\left(j\right)\right)=\exp\left(-\gamma_{m}{\left\|\text{IP}\left(m\left(i\right)\right)-\text{IP}\left(m\left(j\right)\right)\right\|}^{2}\right), \end{array}$$

where *γ*_*m*_ is a parameter used to control the Gaussian kernel bandwidth, and *γ*_*m*_ is defined as follows:

(5) $$\begin{array}{c} \gamma_{m}=\frac{\gamma_{m}^{\prime }}{\left(1/N_{m}\right)\sum_{i=1}^{N_{m}}{\left\|\text{IP}\left(m\left(i\right)\right)\right\|}^{2}}. \end{array}$$

As shown in Formula (5), there is a new bandwidth parameter *γ*_*m*_′, which will be set to 1 according to previous work [34]. Thereafter, a *N*_*m*_ × *N*_*m*_ dimensional miRNA Gaussian interaction profile kernel similarity matrix *KM* can be obtained based on Formula (4).

Similarly, the Gaussian interaction profile kernel similarity between the disease *d*(*i*) and disease *d*(*j*) can be calculated according to the following formulas:

(6) $$\begin{array}{c} KD\left(d\left(i\right),d\left(j\right)\right)=\exp\left(-\gamma_{d}{\left\|\text{IP}\left(d\left(i\right)\right)-\text{IP}\left(d\left(j\right)\right)\right\|}^{2}\right), \end{array}$$

(7) $$\begin{array}{c} \gamma_{d}=\frac{\gamma_{d}^{\prime }}{\left(1/N_{d}\right)\sum_{i=1}^{N_{d}}{\left\|\text{IP}\left(d\left(i\right)\right)\right\|}^{2}}, \end{array}$$

where IP(*d*(*i*)) represents the *i*th row of the adjacency matrix *A*, *γ*_*d*_ is a parameter used to control the Gaussian kernel bandwidth, and *γ*_*d*_′ is a bandwidth parameter that will be set to 1 according to previous work [34]. Hence, a *N*_*d*_ × *N*_*d*_ dimensional disease Gaussian interaction profile kernel similarity matrix *KD* will be obtained based on Formula (6).

### 2.5. Integrated Similarity for miRNAs and Diseases

In this section, in order to improve the accuracy of our prediction results, we will further construct an integrated miRNA similarity matrix *S*_*m*_ and an integrated disease similarity matrix *S*_*d*_ based on these newly obtained matrices such as *FS*, *SS*, *KM*, and *KD* according to the following formulas separately:

(8) $$\begin{array}{c} S_{m}\left(m\left(i\right),m\left(j\right)\right)=\left\{\begin{array}{c} KM\left(m\left(i\right),m\left(j\right)\right),\text{if}\;\;FS\left(i,j\right)=0,\\ \frac{FS\left(m\left(i\right),m\left(j\right)\right)+KM\left(m\left(i\right),m\left(j\right)\right)}{2},\text{otherwise,} \end{array}\right. \end{array}$$

(9) $$\begin{array}{c} S_{d}\left(d\left(i\right),d\left(j\right)\right)=\left\{\begin{array}{c} KD\left(d\left(i\right),d\left(j\right)\right),\text{if}\;\;SS\left(i,j\right)=0,\\ \frac{SS\left(d\left(i\right),d\left(j\right)\right)+KD\left(d\left(i\right),d\left(j\right)\right)}{2},\text{otherwise}. \end{array}\right. \end{array}$$

### 2.6. The Prediction Model of  NBMDA

**Figure 1:**
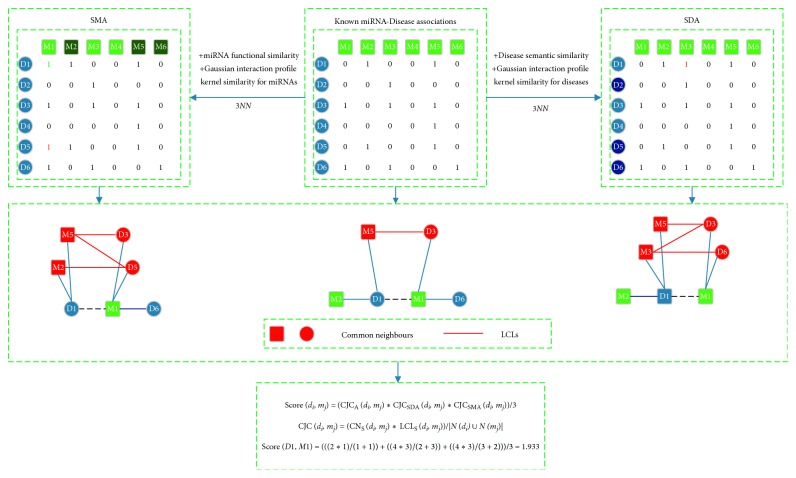
Flowchart of NBMDA.

For a disease node *d*(*i*) and a miRNA node *m*(*j*) in the miRNA-disease association network, according to the concept of common neighbors given in the previous literature [35], considering the computational complexity, we define the common neighbors (*CNs*) between *d*(*i*) and *m*(*j*) as the nodes that are involved in all possible quadrangular closure between *d*(*i*) and *m*(*j*) in the miRNA-disease association network. Obviously, the more *CNs* between two seed nodes such as *d*(*i*) and *m*(*j*), the greater the possibility that these two seed nodes are associated with each other will be. In addition, according to LCP-theory (i.e., the theory of local community paradigm) [35, 36], it is easy to know that the information content related with the common neighbor nodes should be complemented with the topological information emerging from their interactions. Hence, in this section, we introduced LCLs to indicate the number of links that exist between CNs in the miRNA-disease association network. While searching for CNs between two seed nodes in the miRNA-disease association network, we will temporarily remove the connection between these two seed nodes if there is a connection between them. Based on the above LCP-theory, we proposed a novel neighborhood-based computational model called NBMDA for potential miRNA-disease association prediction. In the model of NBMDA, firstly, an integrated miRNA similarity and an integrated disease similarity will be obtained based on the miRNA functional similarity, the disease semantic similarity, and the Gaussian interaction profile kernel similarity for miRNAs and diseases, respectively. And then, considering that known miRNA-disease associations are quite sparse and many diseases (or miRNAs) are associated with only one or a few miRNAs (or diseases), we adopted KNN as a recommendation algorithm to improve the prediction accuracy of NBMDA. Its main idea is to obtain *K* different diseases that are most similar to a randomly given disease *d*(*i*) based on the integrated disease similarity, if most of these *K* diseases are associated with a same miRNA *m*(*k*), then it is obvious that we can assume that disease *d*(*i*) is associated with the miRNA *m*(*k*), and therefore, we can construct a new miRNA-disease association network SDA. In a similar way, we can as well obtain *K* different miRNAs that are most similar to a randomly given miRNA *m*(*j*) based on the integrated miRNA similarity, if most of these *K* miRNAs are associated with a same disease *d*(*k*), then we can assume that miRNA *m*(*j*) is associated with the disease *d*(*k*), and therefore, we can further construct another new miRNA-disease association network SMA. Furthermore, considering the selection of the value of *K* while adopting KNN, we tried different values of *K* from 1 to 5 and found that the best experimental results can be achieved by NBMDA when *K* is set to 3. And as a result, an example is shown in Figure 1, in which, in order to predict the potential association between *D*_1_ and *M*_1_, in SDA, based on the integrated disease similarity, we can obtain three diseases *D*_2_, *D*_5_, and *D6* that are the most similar to *D*_1_, and then, we can obtain a new disease-miRNA association matrix based on 3*NN*. Additionally, in SMA, while calculating the association probability between the seed nodes *D*_1_ and *M*_1_, we will temporarily remove the connection between them.

According to above descriptions, as illustrated in Figure 1, based on the concept of common neighbors and these two newly constructed networks such as SDA and SMA and the original miRNA-disease association network MDA, for any given disease *d*(*i*) and miRNA *m*(*j*), the possibility of potential association between them can be calculated as follows:

(10) $$\begin{array}{c} \text{score}\left(i,j\right)=\frac{{\text{CJC}}_{\text{MDA}}\left(i,j\right)\;*\;{\text{CJC}}_{\text{SDA}}\left(i,j\right)\;*\;{\text{CJC}}_{\text{SMA}}\left(i,j\right)}{3},\\ {\text{CJC}}_{X}\left(i,j\right)=\frac{{\text{CN}}_{\text{S}}\left(X,d\left(i\right),m\left(j\right)\right)\;*\;\left({\text{LCL}}_{\text{S}}\left(X,d\left(i\right),m\left(j\right)\right)\right)}{\left|N\left(X,d\left(i\right)\right)\;\cup \;N\left(X,m\left(j\right)\right)\right|}, \end{array}$$

where *X* ∈ {MDA,SDA,SMA}, CN_S_(*X*, *d*(*i*), *m*(*j*)) represents the number of common neighbors between the disease *d*(*i*) and the miRNA *m*(*j*) in the *X*, LCL_S_(*X*, *d*_*i*_, *m*_*j*_) represents the number of connections existing among the common neighbors between the disease *d*(*i*) and the miRNA *m*(*j*) in the network *X*, *N*(*X*, *d*(*i*)) represents the degree of disease *d*(*i*) in the network *X*, and *N*(*X*, *m*(*j*)) represents the degree of miRNA *m*(*j*) in the network *X*.

## 3. Results

### 3.1. Performance Evaluation

To evaluate the predictive performance of NBMDA, we performed LOOCV and 5-fold CV on dataset1 and LOOCV on dataset2 separately. And during simulation, under the global LOOCV framework, each known miRNA-disease association is alternately utilized as a test sample and other known miRNA-disease associations are considered as a training set. Hence, the process needs to be repeated 5430/250 times in total, and during each round of iteration, the predicted score of test sample was ranked with predicted score of all the miRNA-disease pairs without any known association evidences, and if its ranking is above a given cutoff, it will be considered as a successful prediction made by NBMDA. As for 5-fold CV, all known associations are randomly divided into five equal sized and uncrossed subsets, and among them, four subsets are used for model training and the other for model testing. In order to reduce the impact of data partitioning on the prediction results, we repeated 5-fold CV for 100 times. Finally, the receiver operating characteristic (ROC) curves which show the relationship between the true positive rate (TPR, sensitivity) and false positive rate (FPR, 1-specificity) were drawn for further model evaluation and comparison based on TPR and FPR which were obtained by different thresholds. Here, TPR = TP/(TP + FN) represents the percentage of positive samples identified by the prediction model to all positive samples, and FPR = FP/(FP + TN) indicates the proportion that the prediction model incorrectly considers the negative samples of the positive class to account for all negative samples. Moreover, the areas under ROC curves (AUCs) are calculated as a standard for model performance evaluation. The larger the value of AUC is, the better the prediction performance of the model will be.

**Figure 2:**
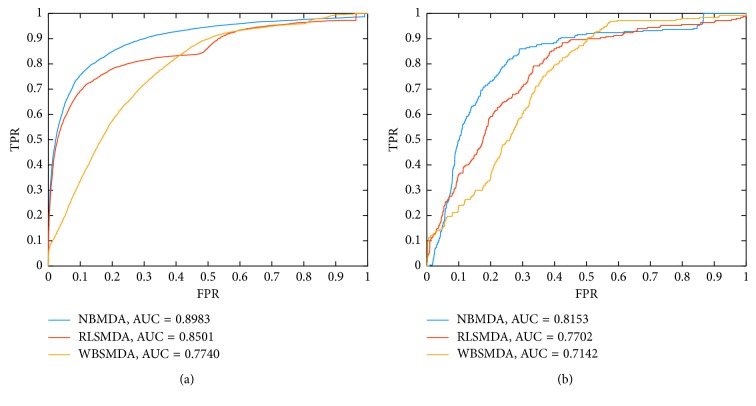
Performance comparisons between NBMDA, RLSMDA, and WBSMDA in global LOOCV. Comparison results based on (a) dataset1 and (b) dataset2.

To further evaluate the predictive performance of NBMDA, we compared NBMDA with two state-of-the-art computational methods such as WBSMDA [37] and RLSMDA [38] in terms of global LOOCV and 5-fold CV as well, and the simulation result is shown in the above Figure 2. It is easy to see that NBMDA, RLSMDA, and WBSMDA can achieve reliable AUCs of 0.8983/0.8153, 0.8501/0.7702, and 0.7740/0.7142, respectively. And moreover, in the 5-fold CV, simulation results show that NBMDA can achieve reliable average AUCs of 0.8975 ± 0.0008. Hence, it is obvious that NBMDA can achieve much better AUCs than these two kinds of state-of-the-art computational methods in terms of both global LOOCV and 5-fold CV, which also demonstrate that NBMDA has reliable performance in potential miRNA-disease association prediction and can improve the predictive performance of previous state-of-the-art computational models effectively.

### 3.2. Case Studies

More and more evidences have shown that miRNAs play an important role in the occurrence and development of human diseases [39, 40]; therefore, effective prediction models are important for discovering potential miRNA-disease associations and medical researches. In order to further evaluate the predictive performance of NBMDA, we implemented the case studies of esophageal neoplasms, breast neoplasms and colon neoplasms on dataset1. The 5430 miRNA-disease associations downloaded from HMDD v2.0 were used as a training sample for model learning, and 50 predicted miRNAs with the strongest association with esophageal neoplasms, breast neoplasms and colon neoplasms will be ranked according to their scores respectively and then verified by the records stored in the dbDEMC and miR2Disease databases. What needs to be emphasized is that only miRNA-disease pairs which have not been included in the HMDD v2.0 database would be considered as validation candidates.

Among these kinds of diseases, esophageal neoplasms (EN) is a cancer with a high incidence and is the eighth most common cancer in the world and the sixth leading cause of cancer death in humans [41]. Esophageal cancer is a common digestive tract cancer, and around 300 thousand people worldwide die from the disease every year. Related data show that the survival rate of patients with esophageal cancer has increased from 15% to 25% in the last five years [42]. If treatment can be performed early in this disease, the survival rate can reach 90%. Therefore, the study of potential biomarkers associated with disease is important for the treatment of related diseases. Recently, related studies have achieved some interesting results. For example, mir-21, miR-143, miR-203, miR-205, and miR-221 were found to be expressed at high levels in esophageal cancer tissues [43]. MiR-375 was found to be expressed at low levels in EN cells, and downregulation of miR-375 can be used as a biomarker for predicting esophageal tumors [44]. In this section, in order to identify miRNAs that are potentially associated with EN, we conducted a case study of EN based on NBMDA, and the simulation results show that 47 out of top 50 potential associated miRNAs are validated by dbDEMC and miR2Disease (Table 1). And moreover, related literatures show that miR-17 (ranking 1st in our prediction list) promotes the growth of EN cells and regulates disorders in a variety of cancers [45, 46]. Mir-125b (ranking 2nd in our prediction list) is expressed at low level in EN cells and can promote the differentiation of EN cells [47, 48].

Breast neoplasms (BN) is a common disease, and breast cancer is the most common malignant tumor in women, accounting for 25% [49, 50]. In recent years, the incidence of breast cancer is on the rise and the mortality rate is decreasing. But in developing countries, the survival rate of patients is still low [51]. In the United States, an average of 185,000 women suffer from breast cancer each year and 44,000 people die from this disease. Therefore, a large number of studies are devoted to the treatment of the disease, and identifying potential miRNAs associated with the disease is an important means. Up to now, a lot of achievements have been made in this direction. For example, miRNA-10b was found to be highly expressed in metastatic breast cancer cells and has a positive regulatory effect on cell migration and invasion [52]. Mir-125b, mir-145, mir-21, and mir-155 are significantly dysregulated in breast cancer patients and can be used as potential biomarkers for early detection of BN [52]. MiR-200c-141, miR-200b-200a-429, and miR-183-96-182 are downregulated in human breast cancer stem cells [53]. Hence, in this section, in order to identify miRNAs that are potentially associated with BN, we conducted a case study of breast neoplasms based on NBMDA. And simulation results show that 10 out of top 10 and 48 out of top 50 predicted miRNAs are validated by dbDEMC and miR2Disease (Table 2). In addition, previously published literatures show that mir-142 (ranking 1st in our prediction list) can regulate the tumorigenicity of human breast cancer stem cells [54]. The expression of miR-150 (ranking 2nd in our prediction list) can significantly inhibit the migration and invasion of breast cancer cells in vitro [55].

Colon neoplasms (CN) are common malignant tumors in the gastrointestinal tract; the incidence rate is second only to gastric cancer and esophageal cancer. It is the most common part of colorectal cancer, accounting for about 60%. In recent years, the number of CN patients is increasing year by year. It is reported that the number of patients with colorectal cancer increase globally by 930,000 per year. However, colon neoplasms is the most easily self-screening conditions, if detected early can be cured. Therefore, detecting potential biomarkers of colon tumors has great significance for the treatment of the disease. Related studies have identified several miRNAs associated with CN. For example, miR-143 and miR-145 are expressed at low levels in CN cells [56]. The serum miRNA-21 content in CN patients is abnormally reduced. Therefore, miRNA-21 may be a potential molecular mechanism regulating angiogenesis in CN [57]. These findings provide a theoretical basis for clinical treatment of CN. Hence, in this section, in order to further discover miRNAs associated with colon tumors, we conducted a case study of colon neoplasms based on NBMDA. And simulation results show that 48 out of top 50 predicted miRNAs are successfully validated by dbDEMC and miR2Disease (Table 3). In addition, some previous experimental literatures show that miR-20a (ranking 1st in our prediction list) upregulates in colon neoplasms cells; its downregulation is also considered to be a biomarker for CN [58]. MiR-18a (ranking 2nd in our prediction list) inhibits the progression of colon cancer and induces apoptosis in colon cancer cells [59]. The miR-143 (ranking 3rd in our prediction list) is downregulated in various cancers including colon cancer, and its antitumor activity can inhibit the proliferation and migration of cancer cells [60].

## 4. Discussion

In this paper, a neighborhood-based computational model called NBMDA is proposed for potential miRNA-disease association prediction. In NBMDA, an integrated disease similarity network and an integrated miRNA similarity network will be constructed first based on the disease semantic similarity, the miRNA functional similarity, and the Gaussian interaction profile kernel similarity for diseases and miRNAs. And then, based on the two integrated similarity networks, the *KNN* method will be adopted as a recommendation algorithm to solve the problem that known miRNA-disease associations are very sparse. Finally, based on the concept of common neighbors, the possibilities of potential associations between miRNAs and diseases can be calculated based on these two newly constructed miRNA-disease association networks and the original miRNA-disease association network. And experimental results show that NBMDA can achieve reliable AUCs of 0.8983/0.8153 and 0.8975 in the frameworks of global LOOCV and 5-fold CV, respectively, which are much better than the AUCs achieved by state-of-the-art prediction models such as WBSMDA and RLSMDA. Moreover, by implementing NBMDA in case studies of esophageal neoplasms, breast neoplasms, and colon neoplasms, there are 47, 48, and 48 out of the top 50 predicted miRNAs having been validated by relevant databases or related literatures separately, which further demonstrate that NBMDA can achieve excellent predictive performance.

The advantages of NBMDA lie in the following aspects: firstly, an integrated disease similarity and an integrated miRNA similarity were obtained by combining the disease semantic similarity, the miRNA functional similarity, and the Gaussian interaction profile kernel similarity for diseases and miRNAs separately, which solved the problem of the sparseness of the similarity matrix to some extent. In addition, the *KNN* method was adopted as a recommendation algorithm to solve the problem of scarcity of known miRNA-disease associations and the problem that the number of common neighbors between two seed nodes in the association network may be 0.

Of course, there will be some limitations in NBMDA that need to be improved in the future works. Firstly, the known miRNA-disease associations obtained from the database are very limited, which accounts for only 2.9% of all possible associations. Secondly, the newly proposed method to solve the problem of the sparseness of the miRNA functional similarity matrix and the disease semantic similarity matrix is relatively simple, and a more effective and reasonable measure of miRNA and disease similarity will be helpful to further improve the prediction performance of NBMDA.

## 5. Conclusions

A growing number of studies have shown that miRNAs are associated with a variety of complex human diseases. Therefore, the detection of potential miRNA-disease associations has great significance for the treatment of the disease and human health. In this paper, we proposed a computational model called NBMDA to discover potential miRNA-disease associations. And in order to evaluate the prediction performance of NBMDA, we compared it with some existing state-of-the-art prediction models in terms of LOOCV and 5-fold CV, respectively, and both the simulation results and case studies indicate that NBMDA can effectively predict potential miRNA-disease associations. Therefore, NBMDA can be a powerful tool for identifying potential biomarkers of diseases.

## Figures and Tables

**Table 1:** The top 50 predicted miRNAs associated with EN obtained by implementing our model NBMDA.

Top 1–25		Top 26–50	
miRNA	Evidence	miRNA	Evidence
hsa-mir-17	dbdemc	hsa-mir-195	dbdemc
hsa-mir-125b	dbdemc	hsa-mir-124	dbdemc
hsa-mir-16	dbdemc	hsa-mir-7	dbdemc
hsa-mir-18a	dbdemc	hsa-mir-93	dbdemc
hsa-mir-29a	dbdemc	hsa-let-7i	dbdemc
hsa-mir-1	dbdemc	hsa-mir-429	dbdemc
hsa-mir-19b	Unconfirmed	hsa-mir-106b	dbdemc
hsa-mir-221	dbdemc	hsa-mir-125a	dbdemc
hsa-mir-200b	dbdemc	hsa-mir-23a	dbdemc
hsa-mir-142	dbdemc	hsa-mir-497	dbdemc
hsa-mir-10b	dbdemc	hsa-mir-106a	dbdemc
hsa-mir-29b	dbdemc	hsa-mir-30c	dbdemc
hsa-let-7e	dbdemc	hsa-mir-151a	dbdemc
hsa-let-7d	dbdemc	hsa-mir-224	dbdemc
hsa-mir-146b	dbdemc	hsa-mir-127	dbdemc
hsa-mir-218	dbdemc	hsa-mir-24	dbdemc
hsa-mir-222	dbdemc	hsa-mir-199b	dbdemc
hsa-mir-133b	dbdemc	hsa-mir-107	dbdemc; miR2Disease
hsa-mir-182	dbdemc	hsa-mir-135a	dbdemc
hsa-mir-181a	dbdemc	hsa-mir-10a	Unconfirmed
hsa-mir-9	dbdemc	hsa-mir-96	dbdemc
hsa-mir-181b	dbdemc	hsa-mir-103a	dbdemc
hsa-mir-30a	dbdemc	hsa-mir-18b	dbdemc
hsa-let-7g	dbdemc	hsa-mir-378a	dbdemc
hsa-let-7f	Unconfirmed	hsa-mir-302b	dbdemc

**Table 2:** The top 50 predicted miRNAs associated with BN obtained by implementing our model NBMDA.

Top 1–25		Top 26–50	
miRNA	Evidence	miRNA	Evidence
hsa-mir-142	dbdemc	hsa-mir-615	dbdemc
hsa-mir-150	dbdemc	hsa-mir-449a	dbdemc
hsa-mir-106a	dbdemc	hsa-mir-1273c	Unconfirmed
hsa-mir-99a	dbdemc	hsa-mir-130b	dbdemc
hsa-mir-98	dbdemc; miR2Dsease	hsa-mir-381	dbdemc
hsa-mir-378a	dbdemc	hsa-mir-542	dbdemc
hsa-mir-130a	dbdemc	hsa-mir-337	dbdemc
hsa-mir-138	dbdemc	hsa-mir-371a	dbdemc
hsa-mir-15b	dbdemc	hsa-mir-95	dbdemc
hsa-mir-532	dbdemc	hsa-mir-181c	dbdemc
hsa-mir-181d	dbdemc; miR2Dsease	hsa-mir-449b	dbdemc
hsa-mir-192	dbdemc	hsa-mir-30e	dbdemc
hsa-mir-198	dbdemc	hsa-mir-637	dbdemc
hsa-mir-186	dbdemc	hsa-mir-519b	dbdemc
hsa-mir-185	dbdemc	hsa-mir-330	dbdemc
hsa-mir-212	dbdemc	hsa-mir-208b	dbdemc
hsa-mir-196b	dbdemc	hsa-mir-372	dbdemc
hsa-mir-527	dbdemc	hsa-mir-602	dbdemc
hsa-mir-526b	dbdemc	hsa-mir-370	dbdemc
hsa-mir-99b	dbdemc	hsa-mir-211	dbdemc; miR2Disease
hsa-mir-1280	Unconfirmed	hsa-mir-32	dbdemc
hsa-mir-574	dbdemc	hsa-mir-514a	dbdemc
hsa-mir-548b	dbdemc	hsa-mir-362	dbdemc
hsa-let-92b	dbdemc	hsa-mir-652	dbdemc
hsa-let-432	dbdemc	hsa-mir-873	dbdemc

**Table 3:** The top 50 predicted miRNAs associated with CN obtained by implementing our model NBMDA.

Top 1–25		Top 26–50	
hsa-mir-20a	Evidence	miRNA	Evidence
hsa-mir-18a	dbdemc; miR2Disease	hsa-mir-200a	dbdemc
hsa-mir-143	dbdemc; miR2Disease	hsa-mir-15a	dbdemc
hsa-mir-19b	dbdemc; miR2Disease	hsa-mir-181a	dbdemc; miR2Disease
hsa-mir-92a	dbdemc; miR2Disease	hsa-mir-181b	dbdemc; miR2Disease
hsa-mir-155	dbdemc	hsa-mir-27a	miR2Disease
hsa-mir-125b	dbdemc; miR2Disease	hsa-mir-133b	dbdemc; miR2Disease
hsa-let-7a	Unconfirmed	hsa-let-7b	dbdemc
hsa-mir-19a	dbdemc; miR2Disease	hsa-mir-142	dbdemc
hsa-mir-16	dbdemc; miR2Disease	hsa-mir-210	dbdemc
hsa-mir-200c	Unconfirmed	hsa-mir-203	dbdemc; miR2Disease
hsa-mir-34a	dbdemc; miR2Disease	hsa-mir-34c	miR2Disease
hsa-let-31	dbdemc; miR2Disease	hsa-mir-100	dbdemc
hsa-let-146a	dbdemc; miR2Disease	hsa-let-7	dbdemc
hsa-mir-10b	dbdemc	hsa-mir-101	dbdemc
hsa-mir-200b	dbdemc; miR2Disease	hsa-mir-106b	dbdemc; miR2Disease
hsa-mir-21	dbdemc	hsa-mir-183	dbdemc; miR2Disease
hsa-mir-218	dbdemc; miR2Disease	hsa-mir-7d	dbdemc
hsa-mir-221	dbdemc	hsa-mir-146b	dbdemc
hsa-mir-182	dbdemc; miR2Disease	hsa-mir-222	dbdemc
hsa-mir-205	dbdemc; miR2Disease	hsa-let-7e	dbdemc
hsa-mir-29a	dbdemc	hsa-let-7c	dbdemc
hsa-mir-1	dbdemc; miR2Disease	hsa-mir-375	dbdemc
hsa-let-9	dbdemc; miR2Disease	hsa-mir-34b	dbdemc; miR2Disease
hsa-let-29b	dbdemc; miR2Disease	hsa-mir-141	dbdemc
hsa-mir-20a	dbdemc; miR2Disease	hsa-mir-150	dbdemc

## Data Availability

Readers can download the dataset used in this article from http://www.cuilab.cn/hmdd and http://www.mir2disease.org/.

## Supplementary Materials

Supplementary Materials Experimentally verified microRNA-disease associations. Each line represents an association between a microRNA and a disease.
